# Cryoprotective Effects of Tuna Skin Antifreeze Peptides on the Quality of Salmon Flesh During Low-Temperature Fluctuations

**DOI:** 10.3390/foods15061105

**Published:** 2026-03-22

**Authors:** Zhe Xu, Ziyu Zhang, Zijin Qin, Tengfei Li, Zihao Zhang, Shuyu Zhou, Jianbo Sun, Tingting Li

**Affiliations:** 1 Key Laboratory of Biotechnology and Bioresources Utilization, College of Life Sciences, Dalian Minzu University, Ministry of Education, Dalian 116600, China; xuzhe@dlnu.edu.cn (Z.X.); 18840172017@163.com (Z.Z.); 17636369272@163.com (T.L.); zzh13562849907@163.com (Z.Z.); 13581008635@163.com (S.Z.); 19841141242@163.com (J.S.); 2 Department of Food Science and Technology, University of Georgia, Clarke, Athens, GA 30602, USA; zq20739@uga.edu

**Keywords:** tuna skin, freeze–thaw cycles, myofibrillar protein, antifreeze peptide, protein oxidation, salmon storage

## Abstract

Repetitive temperature fluctuations during transportation and storage promote ice crystal formation in salmon flesh, leading to protein denaturation, lipid oxidation, and quality loss. Tuna skin, a major by-product of tuna processing, is a potential source of antifreeze peptides (AFPs) but remains underutilized. This study examined the cryoprotective effects of tuna skin-derived AFPs on salmon cubes subjected to repeated freeze–thaw cycles. Cubes treated with AFPs from three groups of protein hydrolysates prepared using trypsin, pepsin, or neutral protease were evaluated for texture, color, water holding capacity (WHC), volatile odor profiles, protein conformation, biochemical indices, and microstructure. AFP treatment improved textural properties, maintained color stability, and reduced thawing, cooking, and centrifugal losses. The neutral protease-treated group exhibited the optimal cryoprotective ability and it also limited aldehyde and sulfide accumulation, preserved the retention rate of α-helix structure at 49% which was higher than 39% in controls, and enhanced Ca^2+^-ATPase activity to 1.75 μmol Pi·mg^−1^·h^−1^ with a 45.8% increase compared to controls, and significantly inhibited protein and lipid oxidation. Microstructural analysis showed compact fibers and intact sarcolemma in the neutral protease-treated group samples, contrasting with severe disruption in controls. This study showed that tuna skin AFPs mitigate freeze–thaw damage in salmon cubes by stabilizing proteins and reducing oxidative deterioration, highlighting their potential as natural, healthy cryoprotectants for seafood preservation, meeting the growing demand of the food industry for clean-label, low-calorie preservation solutions, while advancing the circular economy of aquatic processing via the valorization of tuna skin by-products for high-value seafood applications.

## 1. Introduction

Salmon has a high nutritional value due to its high-quality protein and polyunsaturated fatty acids. Freezing acts as a key method for salmon preservation, suppressing microbial growth and mitigating protein and lipid oxidation [1]. Nevertheless, in the transportation of salmon products, quality changes are relatively common due to prolonged freezing and temperature fluctuations during loading and unloading. Such quality deterioration during freezing and storage is primarily caused by the formation of ice crystals, which disrupt tissue and cellular structures [2]. As a result, the salmon exhibits an altered texture, reduced moisture content, denatured myofibrillar protein (MP), and oxidized lipids, which greatly compromise its nutritional, organoleptic and commercial value.

To solve the problem, cryoprotectants are widely applied to mitigate freezing-induced damage. However, commercial additives such as sucrose and sorbitol have the limitation of high caloric content and excessive sweetness [3], as demonstrated by the fact that each gram of sorbitol provides approximately 2.4 kilocalories (kcal) of energy, while each gram of sucrose provides approximately 4.0 kilocalories (kcal) of energy, which restrict their further application in aquatic products. Therefore, there is growing consumer demand for flavor-neutral cryoprotectants derived from natural polysaccharides and proteins. The studies have shown that AFPs can inhibit ice crystal growth, reduce mechanical damage to muscle cells, and stabilize protein structures through mechanisms such as ice-binding activity and hydrogen-bond interactions with biomolecules [4,5]. These peptides can be obtained by hydrolyzing proteins from food sources derived from diverse aquatic organisms, such as fish scales and fish skin, which are typical by-products of seafood processing often discarded as waste. For instance, AFPs have been successfully extracted from aquatic biomass like grass carp skin and Evynnis japonica (Ej) scales [6,7], and showed significant cryoprotective effects on frozen surimi. However, most existing studies focus on low-value aquatic products such as surimi rather than high-value species like salmon, leaving a critical knowledge gap in understanding AFPs’ efficacy in premium seafood with delicate muscle structures and strict quality requirements. Particularly, AFPs derived from aquatic by-products have gained increasing attention due to their biocompatibility, low toxicity, and potential for sustainable, high-value utilization of processing residues, a potential that translates to a value of roughly 15–20 USD/kg for such bioderived antifreeze agents.

Tuna skin, a major underutilized by-product of tuna processing, serves as an excellent substrate for the production of antifreeze proteins due to its abundance of collagen and various functional proteins, including antioxidant proteins, antibacterial proteins, and antifreeze proteins [8]. Compared to AFPs from other marine by-products such as shrimp heads and fish viscera, tuna skin-derived AFPs offer a distinct advantage in sustainable and abundant raw material supply. Tuna processing generates massive amounts of by-products, accounting for 50% to 70% of the total fish weight [9]. Among these by-products, tuna skin is a key component with a stable proportion, ranging from 4% to 6% of the total fish weight, a considerable share that ensures a continuous and sufficient raw material source for AFP production. This advantage addresses the resource scarcity challenge faced by AFPs derived from other marine by-products with limited overall supply. Previous studies have shown that AFPs derived from marine sources can improve the quality of frozen muscle foods by regulating ice crystal morphology, protecting MPs from denaturation, and inhibiting oxidative reactions. For instance, Ej-AFP peptide from Evynnis japonica scales was reported to control ice crystal sizes by regulating morphology, and also significantly enhanced the WHC of surimi gels after freeze–thaw cycles, with an improvement of more than 40% compared to traditional commercial cryoprotectants [7]. Similarly, antifreeze peptide pk1-A, isolated from Pacific white shrimp head, is effective in preserving snakehead muscle MP integrity during the freeze–thaw process [10], providing preliminary support for the potential protective role of certain marine by-product-derived AFPs in safeguarding the quality of frozen muscle. Despite these advances, research on AFPs derived from tuna skin remains limited, especially regarding their potential role in preserving the quality of high-value species such as salmon.

Therefore, this study aimed to systematically evaluate the cryoprotective effect of tuna skin-derived AFPs on high-value seafood products (using salmon as an example) during freeze–thaw cycles. Textural properties, color, moisture loss, volatile odor compounds, MP conformation, and protein oxidation and denaturation extent were examined to understand the underlying mechanism. These results from the present research offer a scientific rationale for developing naturally derived cryoprotectants in aquatic product storage and promote the sustainable, high-value utilization of tuna processing by-products.

## 2. Materials and Methods

### 2.1. Materials

In this study, salmon flesh was procured from Dalian Rich Foods Co., Ltd. (Dalian, Liaoning, China); the salmon used was of the specific species *Oncorhynchus*, freshly slaughtered and transported to the laboratory under a cold chain at −20 °C; each piece of fish flesh (half a fish) had an average weight of 230 g, while tuna skin was provided by Dalian Global Food Corporation Co., Ltd. (Dalian, Liaoning, China). Neutral protease (Cat#Z8030, 60,000 U/g, tested by casein solution as substrate) and bovine serum albumin (BSA) were sourced from Solarbio Science & Technology Co., Ltd. (Beijing, China), and pepsin (S10029, tested by hemoglobin as substrate) and trypsin (S10032, 1000 U/mg, tested by N-Benzoyl-L-arginine ethyl ester as substrate) were sourced from Shanghai yuanye Bio-Technology Co., Ltd. (Shanghai, China).

### 2.2. Preparation of Hydrolysates from Tuna Skin Protein

All extraction steps were performed under ice bath conditions. The extraction method of tuna skin collagen was carried out with slight modifications according to the protocol described by Sadowska [11].

Frozen tuna skin was thawed in deionized water at room temperature. After thawing, scales, residual meat, and fat were carefully removed. The skin was cut into small pieces and thoroughly rinsed with deionized water. Firstly, the tuna skin was mixed with 0.1 mol/L NaOH solution at a ratio of 1:5 (*w*/*v*) and stirred for 12 h to eliminate non-collagenous components and pigments, with the NaOH solution replaced every 4 h. Subsequently, the tuna skin was repeatedly rinsed with distilled water until neutral pH. For collagen extraction, the processed tuna skin was immersed in 0.5 mol/L acetic acid at a mass ratio of 1:30 (*w*/*v*) and continuously stirred for 48 h.

The mixture of acetic acid and tuna skin was filtered through double-layer gauze, and the filtrate containing solubilized collagen was centrifuged at 10,000× *g* for 30 min to collect the supernatant enriched with crude collagen. Solid sodium chloride was gradually added to the supernatant until no additional white flocculent precipitate was formed. The solution was allowed to stand overnight, followed by centrifugation at 10,000× *g* for 15 min to collect the precipitate. The precipitate was redissolved in 0.5 mol/L acetic acid, and the redissolved solution was dialyzed against 0.1 mol/L acetic acid for 24 h and distilled water for 48 h successively. Finally, the dialysate was freeze-dried (FD-1A-50+, Biocool, Beijing, China) to obtain a spongy collagen solid.

To prepare hydrolysates, deionized water was first used to disperse the spongy collagen solid at a 1:50 (*w*/*v*) ratio, followed by hydrolysis for 5 h using one of three proteases under their respective optimal conditions: neutral protease (50 °C, pH 7.0), trypsin (37 °C, pH 8.0), or pepsin (37 °C, pH 2.0). These three proteases and their respective hydrolysis conditions were selected to cover a broad range of proteolytic environments, where each combination of temperature, pH value and duration represents the enzyme-specific optimal activity range, consistent with the methodologies established in previous studies on collagen hydrolysis [12]. To deactivate the proteases, the enzymatic reactions were brought to an end by 10 min of boiling. Following this, the solution was adjusted to a pH of 7.0, after which centrifugation was performed at 7850× *g* for 10 min at 4 °C to collect the supernatant, which was subsequently freeze-dried. The protein hydrolysates prepared using trypsin, pepsin, or neutral protease were respectively named TH (trypsin hydrolysate), PH (pepsin hydrolysate), and NH (neutral protease hydrolysate). The yield of each hydrolysate was calculated as the mass of freeze-dried product relative to that of initial collagen used, with yields as follows: PH had a yield of 0.4 g per gram of collagen, TH 0.8 g per gram of collagen, and NH 0.85 g per gram of collagen.

### 2.3. Preparatory Treatment of Salmon Cubes

The three-dimensional structure of fish cubes makes them more prone to accumulating ice crystal damage, such as ice crystal growth in intermyofibrillar spaces and transcellular ice crystal formation, and compared with thin slices, more significant differences are observed in their quality deterioration [13].

Salmon flesh underwent deboning, was peeled of their skin, and then diced into 1 × 1 × 1 cm^3^ cubes. The samples were immersed in three peptide solutions (2.0 mg/mL of PH, TH, or NH) at 4 °C for 4 h. The negative control (NCon) was the soaking treatment using bulk deionized water [14]. Following the treatment process, the cubes underwent freezing for 24 h at −25 °C and subsequent thawing for 12 h at 4 °C. Three repetitions of such a freeze–thaw cycle were carried out to replicate extreme commercial storage and transport scenarios [12]. This accelerated test enables comprehensive assessment of the protective effect of the cryoprotectant under challenging conditions. Fresh salmon cubes without freeze–thaw treatment served as the positive control (Con).

### 2.4. Determination During Freeze–Thaw Cycles of Salmon Physical Properties

#### 2.4.1. Textural Profile

To reflect the effect of freeze–thaw cycles on the muscle structure of salmon and evaluate the textural quality by analyzing texture parameters, salmon cubes (1 × 1 × 1 cm^3^) were analyzed for texture using a TA-XT Plus texture analyzer (Stable Micro Systems, Godalming, UK), in accordance with the method described by [15]. Ten replicates per treatment group were examined.

#### 2.4.2. Moisture Variation

Centrifugal loss, thawing loss, and cooking loss were measured to assess the WHC of the samples. Thawing loss was calculated according to Equation (1) [16].

(1) $$\mathrm{Thawing}\;\mathrm{loss}\;\left(\%\right)=\frac{\left(W_{1\;}-{\;W}_{2}\right)}{W_{1}}\;\times \;100\%,$$

where W_1_ and W_2_ denote the cube weights before freezing and after thawing.

To determine centrifugal loss, a 3 g sample was centrifuged at 10,000× *g* for 15 min at 4 °C. The separated water was removed after centrifugation. Centrifugal loss was determined according to Equation (2) [16].

(2) $$\mathrm{Centrifugal}\;\mathrm{loss}\;\left(\%\right)=\frac{\left(W_{3\;}-{\;W}_{4}\right)}{W_{3}}\;\times \;100\%,$$

where W_3_ and W_4_ denote the cube weights prior to and following centrifugation, respectively.

To determine cooking loss, thawed samples were vacuum-packed and heated in a water bath to an internal temperature of 75 °C [17]. Cooking loss was measured according to Equation (3) [17].

(3) $$\mathrm{Cooking}\;\mathrm{loss}\;\left(\%\right)=\frac{\left(W_{5}\;-\;W_{6}\right)}{W_{5}}\;\times \;100\%,$$

where W_5_ and W_6_ denote the cube weights before and after cooking, respectively.

#### 2.4.3. Color Measurement

Color is a visual indicator of fish quality and muscle deterioration, so the color measurement was conducted following freeze–thaw cycles using a CR-400 colorimeter (Konica Minolta, Tokyo, Japan). Specifically, a* (redness), b* (yellowness), and L* (lightness) values were recorded at three different locations on each sample.

#### 2.4.4. Electronic Nose (E-Nose) Analysis

To detect changes in the odor profile of salmon during freeze–thaw cycles, identifying the generation of off-flavors related to quality deterioration, odor assessment was performed with a PEN 3 system (AIRSENSE Analytics GmbH, Schwerin, Germany). Each sample was transferred into a 50 mL hermetically sealed flask and immediately stored in a 4 °C refrigerator until analysis. The carrier gas was supplied at a flow rate of 200 mL/min, and headspace sampling was conducted for 60 s. All analyses were conducted in a fume hood.

### 2.5. Assay of MP Concentration

The MP was isolated according to a prior study with slight adjustments [18]. At a *w*/*v* proportion of 1:4, phosphate-buffered solution (PBS, 20 mM, pH 7.0) was incorporated into the treated salmon sample, followed by thorough mixing. The mixture was subjected to homogenization (T 18 D S25, IKA, Guangzhou, Guangdong, China) at 4000 rpm for 10 min, then centrifugation at 5000× *g* under 4 °C for 15 min. The obtained precipitate underwent the identical process two more times. Subsequently, with PBS (20 mM, pH 6.7, with 0.6 M NaCl), the final precipitate was rinsed and then preserved at 4 °C for subsequent experiments.

MP concentration was quantified by the Biuret assay [19]. Briefly, 2 mL of Biuret reagent was mixed with 0.5 mL of BSA solution ranging in concentration from 0 to 10 mg/mL. The absorbance of the combined solution was determined at 540 nm for standard curve establishment. The absorbance of MP was measured, and using the standard curve, the concentration was calculated.

### 2.6. Analysis of MP Structure

By exploring the effects of freeze–thaw cycles on the secondary and microstructures of MP, the correlation between structural changes and functional deterioration is revealed. At an excitation wavelength of 295 nm and emission over the range of 315–400 nm, fluorescence spectra of MP were measured via a fluorescence spectrometer (Fluorolog-3, Horiba, Jobin Yvon, Longjumeau, France) [20]. Circular dichroism (CD) spectroscopy was conducted to analyze MP secondary structure within the wavelength interval of 200–280 nm, with samples in PBS at 0.2 mg/mL.

The microscopic structure of salmon cubes in the course of freeze–thaw cycles was observed via scanning electron microscopy (SEM) (S-4800, Hitachi Ltd., Tokyo, Japan). Flesh cubes (3 × 3 × 3 mm^3^) underwent fixation in 2.5% glutaraldehyde solution for 24 h, dehydration in a graded ethanol gradient, sputter-coating with gold, and visualization at 5.0 kV with 500× magnification.

### 2.7. Analyses of MP Oxidative Alteration and Lipid Oxidation

#### 2.7.1. Assessment of Surface Hydrophobicity (SoANS)

Hydrophobicity on the surface of MP was determined via 1-anilinonaphthalene-8-sulphonic acid (ANS) as a fluorescent probe [21]. MP solutions (0.2–1.0 mg/mL in 20 mM PBS, pH 6.7, with 0.6 M NaCl) were mixed with ANS solution, and fluorescence intensity was recorded. Fluorescence intensity slope versus protein concentration served as an index of hydrophobicity.

#### 2.7.2. Total Sulfhydryl (T-SH) Content

Quantification of T-SH in MP was determined with a commercial assay kit (A063-2-1, Nanjing Jiancheng Bioengineering Institute Co., Ltd., Nanjing, Jiangsu, China) following the manufacturer’s recommended procedure: 10% tissue homogenate was prepared by homogenizing salmon muscle with 0.9% physiological saline (1:9, *w*/*v*) under an ice bath; the supernatant was collected by centrifugation at 2500 rpm for 10 min; 10 mmol/L standard stock solution, 500 μmol/L standard application solution, and two working solutions (working solution 1: Reagent 1: Reagent 2: Reagent 4 = 50:25:25; working solution 2: Reagent 2: Reagent 3 = 1:75) were prepared as instructed; 10 μL of standard/application solution or sample supernatant was added to corresponding wells followed by 150 μL of working solution, and incubated for 5 min, and absorbance was determined at 405 nm with a microplate analyzer (Bio-Tek, Paramus, NJ, USA); T-SH content was calculated using the kit’s formula.

#### 2.7.3. Solubility

Evaluation of protein solubility was conducted by centrifugation. MP solutions (3 mg/mL) were centrifuged at 4500× *g* under 4 °C for 15 min, and supernatant protein concentration was measured via the Biuret method [19]. Solubility was denoted as

(4) $$\mathrm{Protein}\;\mathrm{solubility}\;\left(\%\right)=\left(\frac{A1}{A2}\right)\;\times \;100\%$$

where A1 is the protein content in the supernatant, and A2 is the total protein content.

#### 2.7.4. Carbonyl Content

Determination of carbonyl content in MP was conducted via a commercial assay kit (A087-1, Nanjing Jiancheng Bioengineering Institute Co., Ltd., Nanjing, Jiangsu, China) and the protocol as described in [22]. Absorbance at 370 nm was measured with a UV spectrophotometer (UV-2450, Mapada Instruments, Shanghai, China) after samples were reacted with 2,4-dinitrophenylhydrazine (DNPH), precipitated with trichloroacetic acid (TCA), and washed with ethanol/ethyl acetate (1:1, *v*/*v*).

#### 2.7.5. Dityrosine Content

With minor adjustments to the method in [23], dityrosine content was assessed. MP solutions underwent centrifugation at 5000× *g* under 4 °C for 5 min, and a fluorescence spectrophotometer (970 CRT, Shanghai Precision & Scientific Instrument Co., Ltd., Shanghai, China) was used to record fluorescence intensity.

#### 2.7.6. Ca^2+^-ATPase Activity

Ca^2+^-ATPase activity of MP was determined with a commercial assay kit (A070-3, Nanjing Jiancheng Bioengineering Institute Co., Ltd., Nanjing, Jiangsu, China) as per the manufacturer’s instructions: 0.02 μmol/mL standard Pi solution (via gradient dilution of 10 mmol/L stock solution), chromogenic agent (Reagent 5A:5B = 7:6), substrate solution (Reagent 1:2:3 = 260:80:80) and Reagent 9 (mixed with its diluent and powder) were prepared; a 10% tissue homogenate was prepared by homogenizing salmon muscle with 0.9% physiological saline (1:9, *w*/*v*) under ice bath, centrifuged at 2500 rpm for 10 min to collect the supernatant, which was then diluted to 1% and quantified for protein concentration via Coomassie Brilliant Blue method (avoiding phosphorus-containing reagents); control and test tubes were set up with samples and reagents, incubated at 37 °C for 10 min, terminated with Reagent 4, and centrifuged to collect the supernatant; the supernatant/standard solution and chromogenic agent were added to blank, standard, control and test tubes, incubated at 37 °C for 5–10 min, and absorbance was determined at 636 nm with a UV spectrophotometer (UV-1600, Mapada Instruments, Shanghai, China) using Milli Q water for baseline correction; Ca^2+^-ATPase activity (μmol Pi/mg/h) was calculated using the kit’s formula.

#### 2.7.7. Sodium Dodecyl Sulfate–Polyacrylamide Gel Electrophoresis (SDS-PAGE)

SDS-PAGE was performed following the protocol reported by Cai et al. with minor modifications [22]. Briefly, a mixture of MP solution (1 mg/mL) and sampling buffer was prepared at a ratio of 4:1, then heated at 100 °C for 10 min using a metal bath. Subsequently, 3 μL of 10 to 250 kDa prestained protein marker ladder (26619, Thermo Fisher Scientific, Waltham, MA, USA) and 10 μL of MP solution were loaded onto 4–12% precast polyacrylamide gels (BDK-PG-41211, Nanjing Biodocon Biotechnology Co., Ltd., Nanjing, Jiangsu, China). After electrophoresis, the gel was stained with Coomassie Brilliant Blue R-250, then destained with a solution containing 30% methanol and 10% acetic acid until distinct protein bands were observed. Gel images were acquired using a luminescence imaging system (Tanon-5200, Shanghai Tianneng Bioengineering Technology Co., Ltd., Shanghai, China).

#### 2.7.8. Identification of Peptide Sequences

The NH fraction with optimal cryoprotective activity was analyzed via liquid chromatography–tandem mass spectrometry (LC-MS/MS) for peptide identification, with in-gel tryptic digestion performed following the classic method with minor modifications [24]. The peptide samples were dissolved in mobile phase A (0.1% formic acid) and separated by an EASY nLC-1200 system (LC140, Thermo Fisher Scientific, Waltham, MA, USA) at a constant flow rate of 400 nL/min, with mobile phase B (0.1% formic acid in 80% acetonitrile) and a gradient program: 2% to 7% B over 1 min, 7% to 35% B over 35 min, 35% to 55% B over 9 min, rising to 100% B in 7 min, and holding at 100% B for the final 8 min. The separated peptides were ionized via a nano-electrospray ionization source and analyzed on a Q Exactive HF-X mass spectrometer (Thermo Fisher Scientific, Waltham, MA, USA), with an electrospray voltage of 2.0 kV, full scan detection at 60,000 resolution, MS/MS analysis at 15,000 resolution with a normalized collision energy of 27%. The data-dependent scan mode with the selection of top 20 most abundant precursor ions and 20 s dynamic exclusion time was used. The obtained MS/MS data were processed using Proteome Discoverer version 2.4 (Thermo Fisher Scientific, Waltham, MA, USA) and searched against the Thunnus protein database. Trypsin/P was specified as the cleavage enzyme allowing up to 2 missed cleavages. The mass tolerance allowed for the precursor ions was 10 ppm, while the mass tolerance of fragment ions is set to 0.02 Da. Carbamidomethyl on cysteine was specified as fixed modification, while oxidation on Methionine and acetyl on protein N-terminal were specified as variable modification. Peptide confidence was set at high.

### 2.8. Statistical Analysis

All experiments were conducted in triplicate. Data were assessed via one-way analysis of variance (ANOVA) coupled with Duncan’s post hoc test, with the significance level established at *p* < 0.05. Statistical analysis was conducted with IBM SPSS 26.0 (IBM Corp., Armonk, NY, USA), and graphical presentation was performed using Origin 2018 (OriginLab, Co., Northampton, MA, USA).

## 3. Results

### 3.1. Textural Properties

Textural properties of salmon samples are shown in Table 1. The fresh salmon sample (Con) exhibited the best performance, as its values for all these textural properties are significantly higher than those of other groups. In contrast, the NCon group (without the addition of tuna skin collagen hydrolysate) showed significant texture degradation after freeze–thaw cycles, with hardness and elasticity decreasing by 59% and 72%, respectively, compared with Con. During the freezing process, ice crystal formation may lead to moisture loss and the disruption of muscle microstructure [25]. SEM observations confirm that tuna skin collagen hydrolysates, especially NH, mitigate ice crystal-mediated damage to salmon muscle microstructure by maintaining densely packed fiber bundles with narrow interfibrillar gaps and intact sarcolemma while NCon shows disorganized, fragmented fibers with enlarged gaps that directly correlate with the graded textural quality across groups.

Of the three treatments (PH, TH, and NH), the NH group exhibited the best textural performance, while TH and PH showed graded improvements relative to the NCon group with the overall protective efficacy following the order NH > TH > PH. Hardness and elasticity in NH were 107% and 173% higher than the NCon group, respectively. In contrast to the other treatment groups, NH treatment generates peptides with a relatively uniform molecular weight distribution as shown in Table S1 and a high proportion of polar amino acids (e.g., aspartic acid, glutamic acid) [26]. These peptides have been reported to inhibit ice crystal nucleation and growth by adsorbing onto crystal surfaces, while simultaneously stabilizing MPs through hydrogen bonding and electrostatic interactions, thereby preserving the integrity of the muscle network [5]. The differences in textural protective efficacy among the three hydrolysates are closely related to their ability to preserve the secondary structure of MPs, especially the α-helix content that is critical for maintaining muscle structural rigidity and network integrity. NH effectively inhibited the α-helix-to-random coil transition during freeze–thaw cycles, retaining a higher α-helix content than TH and PH which directly translated to superior textural properties. Similarly, other texture parameters, including cohesiveness, gumminess, chewiness, and resilience, exhibited consistent improvements with the NH treatment, followed by TH and PH. The NH group showed the highest cohesiveness and gumminess among treatment groups, indicating enhanced structural integrity and resistance to deformation. Additionally, the chewiness and resilience values in the NH group were 72% and 80% higher than those of the NCon group. In other freeze–thaw studies on meat [17], the hardness increase in the optimal treatment group compared to the worst group was approximately 36.2% for the hardness index. In contrast, the hardness increase in our NH group compared to the NCon group reached 106.7%, which is significantly higher. Furthermore, the improvements in other textural indicators in our study were also significantly higher than those in the reference study.

The results of texture analysis showed tuna skin AFPs, particularly NH, effectively mitigate salmon textural degradation during freeze–thaw cycles. Their protective effect is likely attributed to the dual action of suppressing ice crystal damage and stabilizing protein conformation, providing a scientific basis for improving salmon freezing tolerance [27].

### 3.2. Assessment of Physicochemical Characteristics

#### 3.2.1. WHC

Results of thawing, cooking, and centrifugal losses are shown in Table 2. The Con showed highest WHC, with minimal cooking and centrifugal losses of 11.10% and 6.42%, respectively. This was attributed to its intact MP network, which is characterized by a stable tertiary structure (tryptophan (Trp) residues in a hydrophobic microenvironment) and high α-helix content (57%), and which retained water through hydrogen bonding and hydrophobic interactions [27,28]. In contrast, the WHC decreased greatly after freeze–thaw cycles in NCon group. The thawing, cooking, and centrifugal losses increased to 10.91%, 20.08%, and 14.51%, respectively. The degradation in WHC is primarily driven by two factors. One is ice crystal formation and recrystallization during freeze–thaw cycles, which mechanically damage muscle cell membranes and disrupt myofibrillar structures [12,29]. The other is MP denaturation induced by freeze–thaw, which causes structural alterations such as exposure of Trp residues to polar environments and α-helix unfolding to 39% [30].

All peptide-treated groups showed enhanced WHC compared with NCon. In particular, NH showed the strongest effect among treatment groups. The average thawing, cooking, and centrifugal losses were 4.65%, 13.97%, and 8.48%, corresponding to 57.4%, 30.4%, and 41.6% reductions compared with NCon. Although less effective than NH, TH and PH also reduced moisture loss by 41.2% and 21.7% compared with the NCon.

The superior WHC of NH was likely related to its peptide composition. Peptides generated under optimal hydrolysis conditions (50 °C, pH 7.0) exhibited a highly concentrated molecular weight distribution, with details provided in Table S1. Among a total of 869 peptide sequences, 94.8% were concentrated in the range of 702–3000 Da, and 72.7% were even below 2000 Da. These peptides were also rich in polar amino acids such as aspartic acid and glutamic acid [14]. Such peptides not only inhibit ice crystal growth and recrystallization through interfacial adsorption, but also stabilize MP conformation by maintaining the hydrophobic microenvironment of Trp residues and retaining α-helix content (49%) via hydrogen bonding with MP backbones [14]. Through these combined effects, these peptides reduced structural damage and improved the WHC of muscle tissues during freezing and thawing processes [31,32].

#### 3.2.2. Color

LAB color is another crucial sensory attribute influencing consumer preference, characterized by L*, a*, and b* values. a* Value reflects myoglobin content, while b* value could be used to evaluate lipid and carotenoid oxidation extent [33]. In general, the market price of frozen salmon depends largely on its color. As mentioned in previous studies [34], consumers’ willingness to pay (WTP) for salmon increases significantly with improved color characteristics. Salmon with vivid and uniform redness corresponding to optimal a* values and minimal yellowness with low b* values can attract consumers to purchase at a premium. Conversely, salmon with faded redness, excessive yellowness or dull appearance with low L* values is difficult to gain consumer acceptance and even hard to sell at a reasonable price. Therefore, maintaining the surface color of salmon recognized by consumers and the industry is a key factor for the potential application of this study. The result of color parameters is shown in Table 2. Fresh salmon showed the most desirable profile, with L* value of 75.76 (bright, uniform lightness), a* value of 5.55 (vivid redness due to high MP content), and b* value of 12.02 (minimal yellowness due to intact carotenoid retention), indicating the undamaged muscle structures that sequester pigments within cells.

After three freeze–thaw cycles, the NCon group showed obvious color deterioration. All the following changes in color parameters (a*, b*, L*) are compared with the CON group. Its L* value decreased by 29.2% to 53.65, producing a dull, grayish appearance likely caused by the reduced light scatter induced by fiber disruption [35]. The a* value dropped by 77.7% to 1.24, reflecting myoglobin leakage and oxidation (conversion of oxymyoglobin to metmyoglobin) [33]. Meanwhile, b* increased by 64.6% to 19.78, indicating the carotenoid degradation and lipid oxidation induced by intracellular antioxidant loss [36].

Among the tuna skin collagen hydrolysate groups, NH showed the strongest ability to mitigate undesirable color changes. Specifically, NH maintained L* at 67.79 (26.4% higher than NCon and closer to Con), preserved a* at 4.10 (73.9% redness retention, significantly higher than TH at 47.2% and PH at 37.1%), and limited b* elevation to 14.23 (only 18.4% above the Con, compared with increases of 32.4% and 40.3% for TH and PH, respectively). The effective color preservation performance of NH can be attributed to two mechanisms. One mechanism is the inhibition of ice crystal growth and recrystallization, which reduces membrane damage and pigment leakage [30]. The other is the stabilization of myoglobin and carotenoids through hydrogen bonding, which delays oxidative denaturation [32]. These effects not only maintain the visual quality but also support the nutritional integrity of salmon muscle.

### 3.3. Analysis by E-Nose

**Figure 1:**
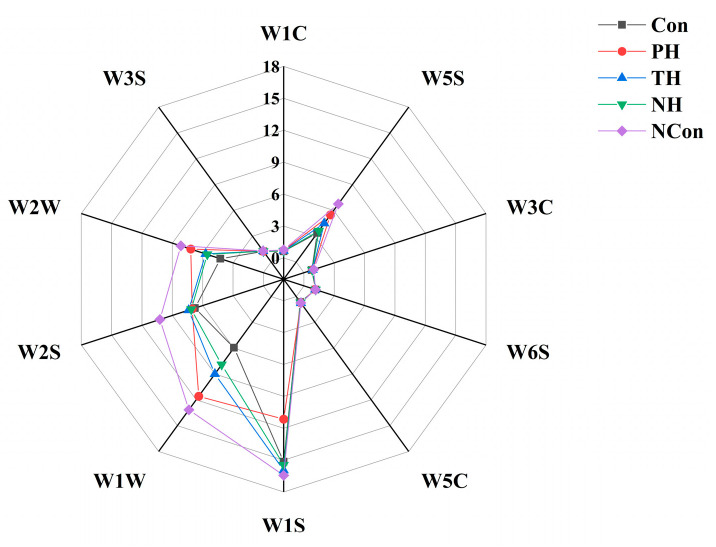
Electronic nose analysis of salmon under different immersion treatments in the course of freeze–thaw cycles.

The result of E-Nose analysis is shown in Figure 1. The E-Nose is a multi-sensor array, which can sensitively detect volatile odor changes in salmon during freeze–thaw cycles. Among the 10 sensors, W1W (sulfides), W2S (alcohols, aldehydes and ketones), W5S (nitrogen oxides), and W1S (methane hydrocarbons) showed the most prominent responses, directly correlating with main fishy odor components [37].

The fresh fish exhibited a compact odor fingerprint, characterized by the lowest responses of W1W, W2S, and W5S, and these collective features represent its natural flavor. Following freeze–thaw cycles, the NCon group exhibited severe odor deterioration, characterized by greatly increased W1W, W1S, and W2S responses, which reflected the accumulation of sulfides (e.g., dimethyl sulfide), hydrocarbon oxidation products, and undesirable alcohols, aldehydes and ketones [38]. It also showed decreased response of W5S, indicating the loss of fresh alcohols and stable nitrogen oxide metabolites [39].

These changes were mainly attributed to freeze–thaw-induced muscle disruption, which activates lipases and proteases and thereby accelerates lipid oxidation and protein degradation.

Among the tuna skin collagen hydrolysate treatments, NH provided the best cryoprotection effect, with its odor fingerprint most closely resembling that of the fresh salmon. Specifically, NH showed notably lower W1S, W1W, and W2S responses, indicating reduced sulfide, hydrocarbon, and undesirable aldehyde, ketone, and alcohol formation, while maintaining a W5S response similar to the Con, reflecting acceptable alcohol retention extent. Mechanistically, NH peptides act through multiple pathways. Firstly, they inhibit ice crystal growth and recrystallization, which reduces muscle damage, limits oxygen–lipid contact, and delays lipid oxidation. Secondly, they stabilize sulfur-containing amino acid precursors (e.g., cysteine), thereby preventing their degradation into volatile sulfides. This latter mechanism is supported by findings in silver carp muscle peptides, which reduced sulfide production by binding sulfur-containing precursors [40].

The E-Nose analysis confirmed that tuna skin AFPs (particularly NH) effectively mitigate freeze–thaw-induced odor deterioration. This efficacy is attributed to their dual role in maintaining muscle integrity and suppressing oxidative and enzymatic reactions, in line with their protective effects on texture and color [27].

### 3.4. MP Structure Changes Analysis

#### 3.4.1. Fluorescence Spectroscopy Analysis

**Figure 2:**
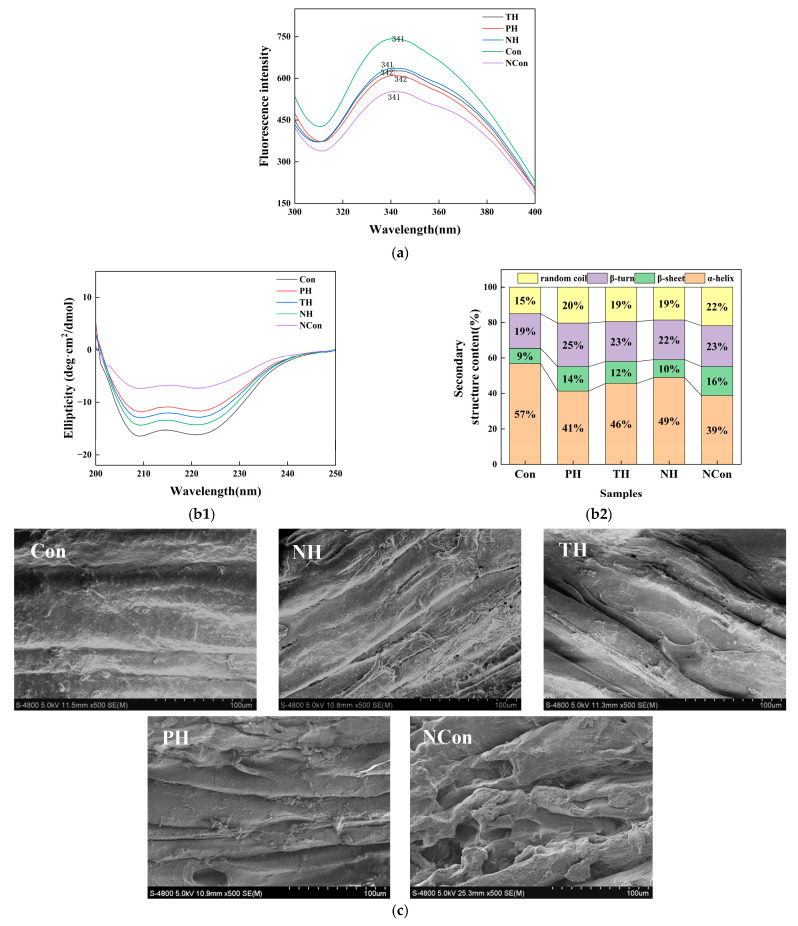
Conformational changes in MPs in salmon cubes under various soaking treatments during freeze–thaw cycling. (**a**) Fluorescence spectra, (**b1**) circular dichroism spectra, (**b2**) secondary structure content, and (**c**) SEM images of salmon (×500).

To assess the tertiary structure of MP, fluorescence spectroscopy was utilized by monitoring the intrinsic fluorescence of Trp residues, whose emission intensity and wavelength reflect the polarity of their microenvironment [41]. As shown in Figure 2a, the Con group exhibited a fluorescence maximum at 341 nm, suggesting the Trp residues were retained in a hydrophobic environment typical of an intact tertiary conformation.

Following freeze–thaw cycles, the NCon group exhibited a marked decrease in fluorescence intensity, while the peak position remained at 340 nm without any noticeable shift. This suggests that Trp residues were exposed to a polar microenvironment due to ice crystal-induced protein unfolding [22]. Among the tuna skin collagen hydrolysate groups, the NH group retained the highest fluorescence intensity at 340 nm, followed by the TH and PH groups. This indicates that NH most effectively maintained the hydrophobic surroundings of Trp residues by inhibiting ice crystal formation and thereby mitigating protein denaturation.

#### 3.4.2. CD Spectroscopy Analysis

CD spectroscopy was employed to analyze the secondary structure of MPs, including α-helices and β-sheets, by detecting the characteristic optical activity of peptide bonds in the far-UV region [42]. Figure 2(b1) shows that the Con group exhibited two distinct negative peaks at 209 nm and 222 nm, corresponding to a high α-helix content that is critical for maintaining the structural rigidity and functional integrity of MPs [43]. Following freeze–thaw cycles, the NCon group exhibited a pronounced flattening of the CD spectrum, indicating extensive α-helix unfolding and secondary structure disruption caused by ice crystal-induced disturbance of intermolecular interactions [29]. In contrast, the spectrum of the NH group most closely resembled that of the Con, indicating effective retention of α-helix structures, whereas the TH and PH groups showed weaker stabilization. The superior performance of NH could be attributed to its peptides forming hydrogen bonds that stabilize α-helix backbones and mitigate the disruption of peptide bonds during freezing.

The quantifications of secondary structure composition of MP in all groups are presented in Figure 2(b2). The Con group exhibited the highest α-helix content (57%), whereas the NH group (49%) was significantly higher than TH (46%), PH (41%), and NCon (39%). This result indicates that NH effectively inhibited the α-helix-to-random coil transition, thereby maintaining a more ordered secondary structure. For random coils, NCon showed the highest proportion (22%), whereas NH exhibited a lower value (19%, comparable to TH) and remained closer to the Con (15%), further supporting the protective role of NH in preserving MP structural integrity.

#### 3.4.3. Correlation with Quality Characteristics

NH effectively protected the α-helix structure and Trp-related hydrophobic microenvironment, which was closely associated with the improved quality of salmon muscle. Preservation of α-helices maintained the MP network integrity and contributed to higher hardness and elasticity, while conformational stabilization enhanced water retention. In contrast, NCon showed markable structural degradation, leading to poor texture and severe moisture loss. Overall, NH peptides preserved salmon quality by stabilizing MP conformation, indicating their molecular cryoprotective effect.

### 3.5. SEM Analysis

SEM enables direct visualization of muscle microstructure, providing insights into freeze–thaw-induced damage to myofibrillar structure and the cryoprotective efficacy of tuna skin collagen hydrolysates. These microstructural observations correlate with macro-quality attributes such as texture, WHC and protein conformational stability (Figure 2c).

The Con showed densely packed muscle fiber bundles with narrow inter-fiber gaps (<10 μm), smooth surfaces, and intact sarcolemma, reflecting an undamaged microstructure that supported its high hardness and elasticity, as well as low moisture loss as described before. These observations confirm that structural integrity is essential for maintaining overall quality stability. In contrast, the NCon group showed significantly disorganized fiber bundles with enlarged gaps (>30 μm), fragmented fibers, and porous surfaces. This structural damage, caused by ice crystal nucleation, growth, and recrystallization, mechanically disrupted sarcolemma and intercellular junctions [44], consistent with NCon’s maximal moisture loss (Section 3.2) and severe protein denaturation (Section 3.4). These results highlight ice crystal-mediated damage as the primary driver of microstructural degradation, which is consistent with the results of [5].

Consistently, the tuna skin collagen hydrolysate groups exhibited a graded protective effect in the order NH > TH > PH. The myofibrillar structural damage in the PH group was the most prominent: its inter-fiber gaps were markedly enlarged to 20–30 μm, accompanied by fiber fractures and the formation of local pores. This outcome stems from its relatively low cryoprotective efficacy. During freeze–thaw cycles, it failed to effectively inhibit the growth and recrystallization of ice crystals, which in turn led to gap expansion, fiber fractures, and pore formation. This also mutually corroborates with the significant disruption of the secondary structure of MPs in the PH group (Section 3.4). The TH group had narrower inter-fiber gaps (15–20 μm) than the PH group but wider than the Con, with slight local fractures. This likely resulted from selective cleavage of arginine and lysine residues abundant in inter-fiber regions, which limited excessive degradation and minimized direct fiber damage [5]. This also explains its superior textural properties relative to PH (Section 3.1). Notably, NH-preserved muscle exhibited narrow interfibrillar gaps (10–15 μm), minimal focal fractures, and intact sarcolemma, closely mirroring the Con. This efficacy arises from NH peptides’ dual mechanisms, suppressing ice crystal growth to mitigate mechanical damage and modulating proteolysis to preserve intermuscular connectivity. Such microstructural integrity directly correlates with NH’s lowest thawing loss (Section 3.2) and superior textural quality (Section 3.1).

SEM observations further reveal that muscle microstructure is governed by a dynamic balance between freeze–thaw-induced ice crystal damage and peptide-mediated protection. In the NCon group, ice crystal-driven fiber fragmentation and gap enlargement directly triggered moisture loss (Section 3.2) and textural degradation (Section 3.1). Conversely, NH sustained fiber compactness via these dual protective mechanisms, thereby preserving salmon’s macro-quality.

### 3.6. Protein Denaturation and Oxidation Analysis

#### 3.6.1. So-ANS

**Figure 3:**
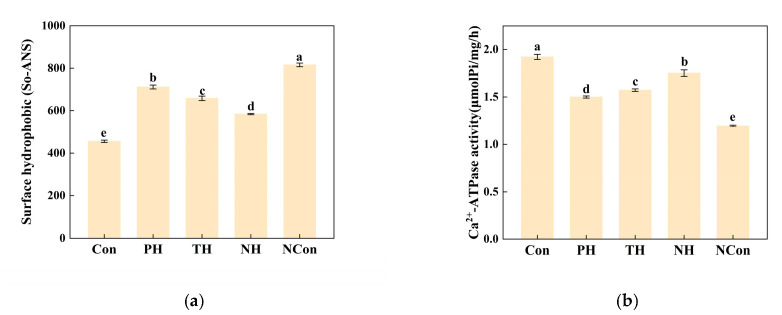

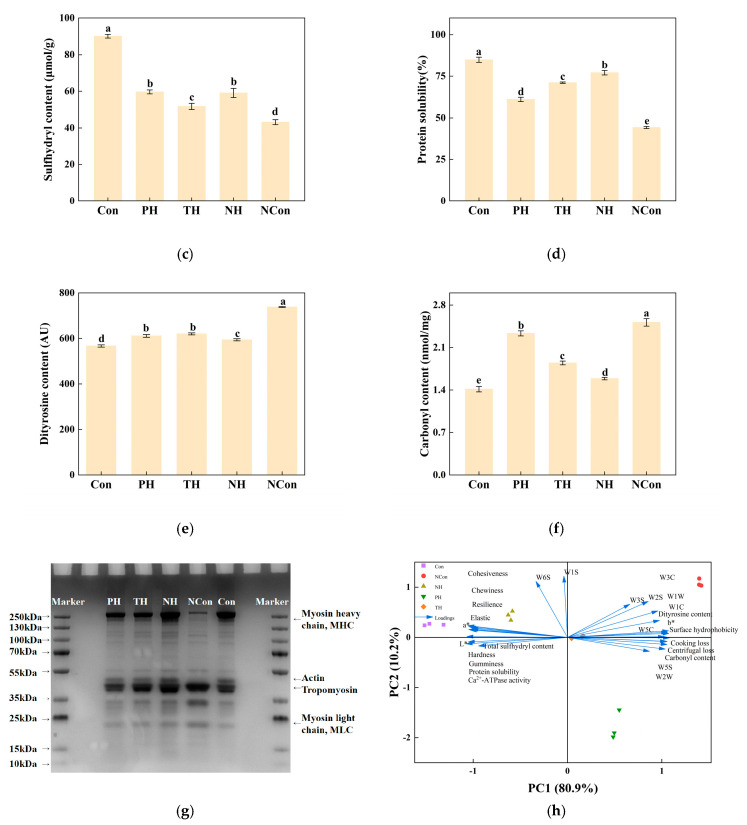
During freeze–thaw cycles, freeze denaturation of salmon MPs and lipids under different immersion solutions. (**a**) Surface hydrophobicity, (**b**) Ca^2+^-ATPase activity, (**c**) total sulfhydryl content, (**d**) protein solubility, (**e**) dityrosine content, (**f**) Carbonyl content, (**g**) SDS-PAGE, and (**h**) PCA. Different letters indicate significant differences (*p* < 0.05).

So-ANS serves as a sensitive protein denaturation indicator and reflects the unveiling of hydrophobic amino acid residues [45]. Figure 3a shows that the NCon group exhibited the maximum So-ANS level (815.66), likely attributed to ice crystal-induced structural damage over repeated freeze–thaw cycles, which promoted oxidation of side chains and exposure of previously buried hydrophobic regions [46].

Among the hydrolysate treatments, the NH group exhibited the lowest So-ANS value (583.67), which was much closer to the Con (455.76) than to the PH (711.42) or TH (657.81) groups, indicating effective mitigation of MP oxidative denaturation and preservation of structural integrity.

#### 3.6.2. Ca^2+^-ATPase Activity

Ca^2+^-ATPase, located in the myosin head, is an indicator of MP structural integrity [26]. Figure 3b shows that the Con exhibited the highest Ca^2+^-ATPase activity (1.92 μmol Pi·mg^−1^·h^−1^), reflecting intact myofibrils and normal myosin functionality, consistent with its high elasticity performance described in Section 3.1. Following freeze–thaw cycles, the NCon group exhibited the lowest activity (1.20 μmol Pi·mg^−1^·h^−1^), which could be attributed to ice crystal-induced disruption of the myosin structure and enzymatic conformation [47], in agreement with its reduced elasticity described in Section 3.1.

Among the hydrolysate treatments, the NH retained the highest Ca^2+^-ATPase activity (1.75 μmol Pi·mg^−1^·h^−1^), surpassing the PH (1.50 μmol Pi·mg^−1^·h^−1^) and TH (1.57 μmol Pi·mg^−1^·h^−1^) groups, thus preserving enzyme functionality and supporting conformational stability (Section 3.4).

#### 3.6.3. T-SH Content

Sulfhydryl (-SH) groups are important for maintaining MP stability, and their content directly reflects the degree of oxidation and denaturation [48]. As shown in Figure 3c, the Con group exhibited the highest T-SH content (90.09 μmol/g), whereas sulfhydryl content of the NCon group decreased to 43.16 μmol/g. This reduction could be attributed to ice crystal damage during freeze–thaw cycles, which exposes -SH groups to oxidation and promotes the formation of disulfide bonds (-S-S-), resulting in aggregation [49,50]. This result is consistent with the increased SoANS described in Section 3.6.1.

Among the hydrolysate treatments, the NH group showed a higher sulfhydryl content (59.05 μmol/g) than the TH group (51.70 μmol/g). Although the value was not significantly different from that of the PH group (59.64 μmol/g) in T-SH content, NH peptides appeared to provide better protection for sulfhydryl groups located in key structural domains, as evidenced by their higher Ca^2+^-ATPase activity, which supports conformational stability as described in Section 3.6.2. In contrast, the TH group showed the lowest sulfhydryl retention, likely because trypsin preferentially cleaves arginine and lysine residues enriched in -SH regions, thereby accelerating oxidative loss.

#### 3.6.4. Solubility of Proteins

Protein solubility reflects the dispersibility of MP in solution and is closely associated with intermolecular interactions and the aggregation state. As shown in Figure 3d, the Con group exhibited the highest solubility (85.02%), with MP remaining stably dispersed to support its high WHC. Conversely, the NCon group exhibited a notable reduction in solubility (44.30%), likely due to freeze–thaw-induced oxidation and aggregation, which aggravated moisture loss described in Section 3.2 [31,51] and contributed to textural deterioration described in Section 3.1.

Among the hydrolysate treatments, the NH group presented the highest solubility (77.08%), attributed to neutral protease-derived peptides mitigating ice crystal damage and moderately hydrolyzing surface segments to reduce aggregation.

#### 3.6.5. Dityrosine Content

Dityrosine is formed through the oxidative crosslinking of tyrosine residues and widely serves as an indicator of MP oxidation [52,53]. As shown in Figure 3e, the Con group exhibited the lowest dityrosine level (566.67 AU), whereas the NCon group reached 738 AU. The increase can be attributed to ice crystal damage during freeze–thaw cycles, which exposes tyrosine residues and accelerates oxidative crosslinking, accompanied by elevated carbonyl formation as described previously [23].

Among the hydrolysate treatments, the NH group showed the lowest dityrosine level (594 AU), followed by the PH (611 AU) and TH (620 AU) groups, due to the mild hydrolytic action of neutral protease (reducing tyrosine activation) and peptides limiting oxidation-prone residue exposure.

#### 3.6.6. Carbonyl Content

Carbonyl (-CO-) content is used to determine MP oxidation extent, reflecting irreversible structural damage. As shown in Figure 3f, the Con group exhibited the lowest carbonyl level (1.39 nmol/mg), whereas the NCon group reached 2.56 nmol/mg. This increase can be attributed to both enzymatic and non-enzymatic oxidation occurring during freeze–thaw cycles, in which residues such as lysine and arginine are converted into carbonyl derivatives, thereby aggravating structural disorder [54,55].

Among the hydrolysate-treated groups, the NH sample displayed the lowest carbonyl content (1.61 nmol/mg), likely associated with NH-derived peptides stabilizing MP conformation and limiting oxidizable residue exposure.

#### 3.6.7. SDS-PAGE

As shown in Figure 3g, the main visible bands include myosin heavy chain (MHC, 200 kDa), actin (42 kDa), tropomyosin (36 kDa) and myosin light chain (MLC, 0–30 kDa). All samples exhibit typical MP bands, verifying reliable MP extraction. Con without freezing presents clear, sparse bands with sharp, non-diffused MHC, actin, tropomyosin and trace natural MLC, reflecting native MP structure. NCon without hydrolysate protection shows significant protein degradation, with band broadening, tailing, diffusion, intensified low-molecular-weight bands and nearly vanished MHC. These changes indicate severe MP degradation, fragmentation, reduced partial protein solubility and impaired structural integrity induced by freezing. Compared with NCon, all the hydrolysate-treated groups show varying protective effects, characterized by intact MHC and regular overall band distribution, confirming hydrolysates effectively inhibit freezing-induced MP structural damage. NH group exerts the most prominent protective effect, with its electrophoretic profile closest to that of Con, intact MHC free of obvious degradation, fewer low-molecular-weight bands and MP composition highly consistent with the native state. TH and PH groups also alleviate the formation of low-molecular-weight degradation bands, but their protective effects are lower than those of the NH group.

#### 3.6.8. Identification and Characterization of Peptides in the NH Fraction

To clarify the material basis of the antifreeze activity of the NH fraction, the fraction was subjected to LC-MS/MS-based peptidomic analysis with detailed data summarized in Supplementary Table S1. In terms of molecular weight distribution, the identified peptides exhibited a highly concentrated range of 702–5123 Da, among which 623 peptides (71.7%) had a molecular weight of 2000 Da or less and 824 peptides (94.8%) fell into the range of 702–3000 Da. This low-molecular-weight feature is conducive to the adsorption of peptides onto ice crystal surfaces and serves as a typical structural prerequisite for exerting cryoprotective effects. Regarding peptide sequence characteristics, the 869 peptides were enriched in polar amino acids such as aspartic acid (Asp) and glutamic acid (Glu), and these polar groups can form hydrogen bonds with water molecules to inhibit ice crystal growth, thereby further supporting the antifreeze activity of the NH fraction [56].

#### 3.6.9. Principal Component Analysis (PCA)

The PCA biplot is shown in Figure 3h, constructed with key physicochemical, structural and biochemical variables across all experimental groups, and it visualizes group distribution and inter-variable associations. The first two principal components explained 80.9% of total variance with PC1 as the horizontal axis and 10.2% of total variance with PC2 as the vertical axis, collectively contributing to 91.1% of the dataset’s core variation. Clear systematic group separation was observed. Con clustered in the negative region of PC1, aligning with protein quality indicators such as Ca^2+^-ATPase activity, protein solubility and T-SH content. NCon was localized to the positive region of PC1, and associated with oxidative and structural damage indices such as W2S, dityrosine content and SoANS. On the PC1 axis, NH was positioned between Con and NCon, though it clustered more closely with Con. PH was located in the lower-right quadrant, characterized by positive PC1 and negative PC2 values. TH was also positioned between the two extreme groups. Clustered loading arrows revealed strong positive correlations between paired variables, such as W2S and dityrosine, Ca^2+^-ATPase activity and protein solubility. This biplot enabled a more intuitive and integrated interpretation of group differences and variable relationships than single-variable comparisons, confirming that NH exerted the most comprehensive MP protective effect. NCon showed severe protein oxidative denaturation from freeze–thaw damage, while PH and TH presented intermediate changes, verifying that tuna skin AFPs, especially the NH fraction, effectively maintain muscle protein stability during freeze–thaw cycles, supporting their application in frozen preservation of aquatic products.

## 4. Conclusions

For AFPs derived from tuna skin collagen hydrolysates prepared with pepsin, trypsin, and neutral protease, NH exhibited the most effective cryoprotective performance. It effectively maintained the texture, color, and WHC of salmon samples after freeze–thaw cycles. NH also reduced MP aggregation and oxidation, and stabilized protein conformation. For example, NH’s α-helix content (49%) was the highest among all treatment groups except Con. These protective effects are likely ascribed to the suppression of ice crystal growth and the preservation of protein structural integrity. Notably, NH overcomes key limitations of commercial cryoprotectants including high caloric content, potential off-flavors, and consumer concerns over synthetic additives. As a natural derivative of tuna skin, which is an underutilized seafood processing waste, NH offers a safe and biocompatible alternative that aligns with circular economy principles. This aspect has not received sufficient attention in previous studies on AFPs derived from marine by-products. In conclusion, NH showed strong cryoprotective potential and represents a promising natural preservative for aquatic products. This study provides a viable alternative to synthetic cryoprotectants and establishes a sustainable approach for valorizing tuna processing by-products. It offers practical guidance for the food industry in pursuing natural, safe and resource-efficient preservation technologies.

## Figures and Tables

**Table 1:** Texture of salmon cubes under different treatment solutions in the course of freeze–thaw cycles.

Samples	Hardness (g)	Elasticity (mm)	Cohesiveness	Gumminess (g)	Chewiness (mJ)	Resilience (mm)
Con	14.49 ± 0.97 ^a^	1.70 ± 0.21 ^a^	1.81 ± 0.08 ^a^	11.35 ± 0.58 ^a^	0.53 ± 0.05 ^a^	0.36 ± 0.01 ^a^
PH	7.20 ± 0.42 ^d^	0.73 ± 0.07 ^cd^	0.72 ± 0.06 ^d^	6.19 ± 0.57 ^d^	0.30 ± 0.03 ^cd^	0.18 ± 0.01 ^cd^
TH	9.86 ± 0.62 ^c^	0.96 ± 0.05 ^c^	0.94 ± 0.06 ^c^	7.29 ± 0.48 ^c^	0.36 ± 0.02 ^bc^	0.19 ± 0.01 ^c^
NH	12.40 ± 0.47 ^b^	1.31 ± 0.33 ^b^	1.45 ± 0.10 ^b^	8.83 ± 0.52 ^b^	0.43 ± 0.05 ^b^	0.27 ± 0.04 ^b^
NCon	6.00 ± 0.67 ^e^	0.48 ± 0.03 ^d^	0.54 ± 0.07 ^e^	5.43 ± 0.38 ^d^	0.25 ± 0.05 ^d^	0.15 ± 0.02 ^d^

Notes: The letters (a–e) in the same column indicate significant differences (*p* < 0.05) among samples, and the values are given as the mean ± standard error (*n* = 10). Abbreviation: Con, positive control; NH, tuna skin neutral protease hydrolysate; TH, tuna skin trypsin hydrolysate; PH, tuna skin pepsin hydrolysate; NCon, negative control.

**Table 2:** Moisture loss and color of salmon cubes under different treatment solutions in the course of freeze–thaw cycles.

Samples	L*	a*	b*	Thawing Loss (%)	Cooking Loss (%)	Centrifugal Loss (%)
Con	75.76 ± 3.02 ^a^	5.55 ± 0.89 ^a^	12.02 ± 0.83 ^d^	--	11.10 ± 0.66 ^d^	6.42 ± 0.52 ^e^
PH	59.98 ± 0.57 ^c^	2.06 ± 0.36 ^cd^	16.86 ± 0.87 ^b^	8.54 ± 0.59 ^b^	16.51 ± 0.58 ^b^	12.43 ± 0.50 ^b^
TH	60.81 ± 0.96 ^c^	2.62 ± 0.21 ^c^	15.92 ± 0.57 ^b^	6.42 ± 0.49 ^c^	16.00 ± 0.52 ^b^	10.40 ± 1.28 ^c^
NH	67.79 ± 0.23 ^b^	4.10 ± 0.41 ^b^	14.23 ± 0.40 ^c^	4.65 ± 0.29 ^d^	13.97 ± 0.20 ^c^	8.48 ± 0.69 ^d^
NCon	53.65 ± 0.85 ^d^	1.24 ± 0.10 ^d^	19.78 ± 0.85 ^a^	10.91 ± 0.83 ^a^	20.08 ± 0.63 ^a^	14.51 ± 0.55 ^a^

Notes: The letters (a–e) in the same column denote significant differences (*p* < 0.05) among samples, and the values are given as the mean ± standard error (*n* = 3). Abbreviation: Con, positive control; NH, tuna skin neutral protease hydrolysate; TH, tuna skin trypsin hydrolysate; PH, tuna skin pepsin hydrolysate; NCon, negative control.

## Data Availability

The original contributions presented in the study are included in the article, further inquiries can be directed to the corresponding author.

## Supplementary Materials

The following supporting information can be downloaded at https://www.mdpi.com/article/10.3390/foods15061105/s1, Table S1. Detailed peptidomic data of the NH fraction identified by LC-MS/MS analysis.

## Abbreviations

The following abbreviations are used in this manuscript:

MP	Myofibrillar protein
Con	Positive control
NCon	Negative control
NH	Tuna skin neutral protease hydrolysate
TH	Tuna skin trypsin hydrolysate
PH	Tuna skin pepsin hydrolysate
SEM	Scanning electron microscopy
AFPs	Antifreeze peptides
ANOVA	Analysis of variance
ANS	1-anilinonaphthalene-8-sulphonic acid
PBS	Phosphate-buffered solution
BSA	Bovine serum albumin
CD	Circular dichroism
DNPH	2,4-Dinitrophenylhydrazine
E-nose	Electronic nose
T-SH	Total sulfhydryl
SoANS	Surface hydrophobicity
TCA	Trichloroacetic acid
WHC	Water holding capacity
SDS-PAGE	Sodium dodecyl sulfate–polyacrylamide gel electrophoresis
LC-MS/MS	Liquid chromatography–tandem mass spectrometry
PCA	Principal Component Analysis
Ej	Evynnis japonica
MHC	myosin heavy chain
MLC Trp	myosin light chain tryptophan

## References

[1] Banerjee R., Maheswarappa N.B. (2019). Superchilling of muscle foods: Potential alternative for chilling and freezing. Crit. Rev. Food Sci. Nutr..

[2] Zhang M., Haili N., Chen Q., Xia X., Kong B. (2018). Influence of ultrasound-assisted immersion freezing on the freezing rate and quality of porcine longissimus muscles. Meat Sci..

[3] Walayat N., Xiong H., Xiong Z., Moreno H.M., Nawaz A., Niaz N., Randhawa M.A. (2020). Role of cryoprotectants in surimi and factors affecting surimi gel properties: A review. Food Rev. Int..

[4] Chen X., Li X., Yang F., Wu J., Huang D., Huang J., Wang S. (2022). Effects and mechanism of antifreeze peptides from silver carp scales on the freeze-thaw stability of frozen surimi. Food Chem..

[5] Chen X., Wu J., Li X., Yang F., Yu L., Li X., Huang J., Wang S. (2022). Investigation of the cryoprotective mechanism and effect on quality characteristics of surimi during freezing storage by antifreeze peptides. Food Chem..

[6] Peng X., Mei J., Zhao L., Wei Y., Liu K., Zhong S., Hu J., Yuan M. (2025). Investigation of the protective effects of antifreeze peptides from grass carp skin on frozen surimi. LWT-Food Sci. Technol..

[7] Jiang W., Yang F., Cai D., Du J., Wu M., Cai X., Chen X., Wang S. (2025). Peptidomics & molecular simulation-based specific screening of antifreeze peptides from *Evynnis japonica* scale and the action mechanism. J. Agric. Food Chem..

[8] Ahmed R., Chun B.-S. (2018). Subcritical water hydrolysis for the production of bioactive peptides from tuna skin collagen. J. Supercrit. Fluids.

[9] Oliveira D., Bernardi D., Drummond F., Dieterich F., Boscolo W., Leivas C., Kiatkoski E., Waszczynskyj N. (2017). Potential use of tuna (*Thunnus albacares*) by-product: Production of antioxidant peptides and recovery of unsaturated fatty acids from tuna head. Int. J. Food Eng..

[10] Tan M., Han M., Zhou Y., Chen Z., Cao W. (2025). Trash to treasure: Potential antifreeze peptide from *Litopenaeus vannamei* head via ultrasound-assisted autolysis. Food Chem. X.

[11] Sadowska M., Kołodziejska I., Niecikowska C. (2003). Isolation of collagen from the skins of Baltic cod (*Gadus morhua*). Food Chem..

[12] Zhu K., Zheng Z., Dai Z. (2022). Identification of antifreeze peptides in shrimp byproducts autolysate using peptidomics and bioinformatics. Food Chem..

[13] Egelandsdal B., Abie S.M., Bjarnadottir S., Zhu H., Kolstad H., Bjerke F., Martinsen O.G., Mason A., Munch D. (2019). Detectability of the degree of freeze damage in meat depends on analytic-tool selection. Meat Sci..

[14] Xu Z., Cao S., Cui N., Zhang R., Qin Z., Liu H., Wu J., Du M., Tan Z., Li T. (2025). Screening and characterization of an antifreeze peptide from sea cucumber intestinal protein hydrolysates. Food Chem..

[15] Cai L., Nian L., Zhao G., Zhang Y., Sha L., Li J. (2018). Effect of herring antifreeze protein combined with chitosan magnetic nanoparticles on quality attributes in red sea bream (*Pagrosomus major*). Food Bioprocess Technol..

[16] Du X., Chang P., Tian J., Kong B., Sun F., Xia X. (2020). Effect of ice structuring protein on the quality, thermal stability and oxidation of mirror carp (*Cyprinus carpio* L.) induced by freeze-thaw cycles. LWT-Food Sci. Technol..

[17] Li Y., Han X., Zhang Y., Wang Y., Wang J., Teng W., Wang W., Cao J. (2024). Thawed drip and its membrane-separated components: Role in retarding myofibrillar protein gel deterioration during freezing-thawing cycles. Food Res. Int..

[18] Zhang K., Wang Y., Fan X., Li N., Tan Z., Liu H., Liu X., Zhou D., Li D. (2024). Effects of calcium chloride on the gelling and digestive characteristics of myofibrillar protein in *Litopenaeus vannamei*. Food Chem..

[19] Gornall A.G., Bardawill C.J., David M.M. (1949). Determination of serum proteins by means of the biuret reaction. J. Biol. Chem..

[20] Ni X., Chen C., Li R., Liu Q., Duan C., Wang X., Xu M. (2024). Effects of ultrasonic treatment on the structure and functional characteristics of myofibrillar proteins from black soldier fly. Int. J. Biol. Macromol..

[21] Lu H., Zhang L., Li Q., Luo Y. (2016). Comparison of gel properties and biochemical characteristics of myofibrillar protein from bighead carp (*Aristichthys nobilis*) affected by frozen storage and a hydroxyl radical-generation oxidizing system. Food Chem..

[22] Cai L., Nian L., Cao A., Zhang Y., Li X. (2019). Effect of carboxymethyl chitosan magnetic nanoparticles plus herring antifreeze protein on conformation and oxidation of myofibrillar protein from red sea bream (*Pagrosomus major*) after freeze-thaw treatment. Food Bioprocess Technol..

[23] Davies K.J., Delsignore M.E. (1987). Protein damage and degradation by oxygen radicals. III. Modification of secondary and tertiary structure. J. Biol. Chem..

[24] Mann A.S.M.W.O.V.M. (1996). Mass spectrometric sequencing of proteins from silver-stained polyacrylamide gels. Anal. Chem..

[25] Xie J., Yan Y., Pan Q., Shi W., Gan J., Lu Y., Tao N., Wang X., Wang Y., Xu C. (2020). Effect of frozen time on *Ctenopharyngodon idella* surimi: With emphasis on protein denaturation by Tri-step spectroscopy. J. Mol. Struct..

[26] Yasemi M. (2017). Prevention of denaturation of freshwater crayfish muscle subjected to different freeze-thaw cycles by gelatin hydrolysate. Food Chem..

[27] Wang W., Bu Y., Li W., Zhu W., Li J., Li X. (2024). Effects of nano freezing-thawing on myofibrillar protein of Atlantic salmon fillets: Protein structure and label-free proteomics. Food Chem..

[28] Nikoo M., Benjakul S., Ahmadi Gavlighi H., Xu X., Regenstein J.M. (2019). Hydrolysates from rainbow trout (*Oncorhynchus mykiss*) processing by-products: Properties when added to fish mince with different freeze-thaw cycles. Food Biosci..

[29] Zhu X., Yuan P., Zhang T., Wang Z., Cai D., Chen X., Shen Y., Xu J., Song C., Goff D. (2022). Effect of carboxymethyl chitosan on the storage stability of frozen dough: State of water, protein structures and quality attributes. Food Res. Int..

[30] Huff-Lonergan E., Lonergan S.M. (2005). Mechanisms of water-holding capacity of meat: The role of postmortem biochemical and structural changes. Meat Sci..

[31] Zhang Z., Xiong Z., Lu S., Walayat N., Hu C., Xiong H. (2020). Effects of oxidative modification on the functional, conformational and gelling properties of myofibrillar proteins from *Culter alburnus*. Int. J. Biol. Macromol..

[32] Cao L., Majura J.J., Liu L., Cao W., Chen Z., Zhu G., Gao J., Zheng H., Lin H. (2023). The cryoprotective activity of tilapia skin collagen hydrolysate and the structure elucidation of its antifreeze peptide. LWT-Food Sci. Technol..

[33] Lan W., Hu X., Sun X., Zhang X., Xie J. (2019). Effect of the number of freeze-thaw cycles number on the quality of Pacific white shrimp (*Litopenaeus vannamei*): An emphasis on moisture migration and microstructure by LF-NMR and SEM. Aquac. Fish..

[34] Steine G., Alfnes F., Rørå M.B. (2005). The effect of color on consumer WTP for farmed salmon. Mar. Resour. Econ..

[35] Hopkins D.L., Littlefield P.J., Thompson J.M. (2000). A research note on factors affecting the determination of myofibrillar fragmentation. Meat Sci..

[36] Bindu J., Ginson J., Kamalakanth C.K., Asha K.K., Srinivasa Gopal T.K. (2013). Physico-chemical changes in high pressure treated indian white prawn (*Fenneropenaeus indicus*) during chill storage. Innov. Food Sci. Emerg. Technol..

[37] Liu S., Liao T., McCrummen S.T., Hanson T.R., Wang Y. (2016). Exploration of volatile compounds causing off-flavor in farm-raised channel catfish (*Ictalurus punctatus*) fillet. Aquac. Int..

[38] Vidal N.P., Manzanos M.J., Goicoechea E., Guillen M.D. (2016). Farmed and wild sea bass (*Dicentrarchus labrax*) volatile metabolites: A comparative study by SPME-GC/MS. J. Sci. Food Agric..

[39] Chung H.Y., Yung I.K.S., Ma W.C.J., Kim J.-S. (2001). Analysis of volatile components in frozen and dried scallops (*Patinopecten yessoensis*) by gas chromatography/mass spectrometry. Food Res. Int..

[40] Cui M., Li J., Li J., Wang F., Li X., Yu J., Huang Y., Liu Y. (2023). Screening and characterization of a novel antifreeze peptide from silver carp muscle hydrolysate. Food Chem..

[41] Walayat N., Xiong Z., Xiong H., Moreno H.M., Niaz N., Ahmad M.N., Hassan A., Nawaz A., Ahmad I., Wang P.K. (2020). Cryoprotective effect of egg white proteins and xylooligosaccharides mixture on oxidative and structural changes in myofibrillar proteins of *Culter alburnus* during frozen storage. Int. J. Biol. Macromol..

[42] Tang J., Yang C., Zhou L., Ma F., Liu S., Wei S., Zhou J., Zhou Y. (2012). Studies on the binding behavior of prodigiosin with bovine hemoglobin by multi-spectroscopic techniques. Spectrochim. Acta Part A Mol. Biomol. Spectrosc..

[43] Zhao W., Yang R. (2008). The effect of pulsed electric fields on the inactivation and structure of lysozyme. Food Chem..

[44] Nian L., Cao A., Cai L. (2020). Investigation of the antifreeze mechanism and effect on quality characteristics of largemouth bass (*Micropterus salmoides*) during F-T cycles by hAFP. Food Chem..

[45] Shi Y., Li R., Tu Z., Ma D., Wang H., Huang X., He N. (2015). Effect of γ-irradiation on the physicochemical properties and structure of fish myofibrillar proteins. Radiat. Phys. Chem..

[46] Zhang Y., Kim Y.H.B., Puolanne E., Ertbjerg P. (2022). Role of freezing-induced myofibrillar protein denaturation in the generation of thaw loss: A review. Meat Sci..

[47] Yang F., Jiang W., Chen X., Wu J., Huang J., Cai X., Wang S. (2023). Investigation on the quality regulating mechanism of antifreeze peptides on frozen surimi: From macro to micro. Food Res. Int..

[48] Li N., Meng Q., Zhao X., Yin F., Zhou D., Li D. (2026). Pressure-driven depolymerization of Antarctic krill aggregates: Structural reconfiguration and gelation-enhanced functionality. Food Chem..

[49] Chen S., Tao F., Pan C., Hu X., Ma H., Li C., Zhao Y., Wang Y. (2020). Modeling quality changes in Pacific white shrimp (*Litopenaeus vannamei*) during storage: Comparison of the Arrhenius model and Random Forest model. J. Food Process. Preserv..

[50] Li F., Du X., Wang B., Pan N., Xia X., Bao Y. (2021). Inhibiting effect of ice structuring protein on the decreased gelling properties of protein from quick-frozen pork patty subjected to frozen storage. Food Chem..

[51] Benjakul S., Visessanguan W., Thongkaew C., Tanaka M. (2005). Effect of frozen storage on chemical and gel-forming properties of fish commonly used for surimi production in Thailand. Food Hydrocoll..

[52] Colombo G., Clerici M., Giustarini D., Portinaro N., Badalamenti S., Rossi R., Milzani A., Dalle-Donne I. (2015). A central role for intermolecular dityrosine cross-linking of fibrinogen in high molecular weight advanced oxidation protein product (AOPP) formation. Biochim. Et Biophys. Acta.

[53] Ma J., Wang X., Li Q., Zhang L., Wang Z., Han L., Yu Q. (2021). Oxidation of myofibrillar protein and crosslinking behavior during processing of traditional air-dried yak (*Bos grunniens*) meat in relation to digestibility. LWT-Food Sci. Technol..

[54] Nikoo M., Benjakul S. (2015). Potential application of seafood-derived peptides as bifunctional ingredients, antioxidant–cryoprotectant: A review. J. Funct. Foods.

[55] Lorido L., Ventanas S., Akcan T., Estevez M. (2016). Effect of protein oxidation on the impaired quality of dry-cured loins produced from frozen pork meat. Food Chem..

[56] Xu Z., Guan S., Cao S., Zhang R., Liu H., Qin Z., Dong X., Wu J., Li T. (2025). Cryoprotective activity of different characterized fractions isolated from sea cucumber intestinal protein hydrolysates against salmon. Food Chem..

